# POSoligo software for in vitro gene synthesis

**DOI:** 10.1038/s41598-024-59497-3

**Published:** 2024-05-15

**Authors:** Yingying Tong, Jie Sun, Yang Chen, Changhua Yi, Hua Wang, Caixin Li, Nana Dai, Guanghua Yang

**Affiliations:** 1 International Research Center for Biological Sciences, Ministry of Science and Technology, Shanghai Ocean University, Shanghai, 201306 China; 2 National Aquatic Animal Pathogen Collection Center, Shanghai Ocean University, Shanghai, 201306 China; 3 Aquatic Animal Genetics and Breeding Center, Shanghai Ocean University, Shanghai, 201306 China; 4 Shanghai Telebio Biomedical Technology Co., LTD, Shanghai, China; 5 Anhui Health College, Chizhou, 247100 Anhui China

**Keywords:** Nucleotide synthesis, Patch oligonucleotide synthesis, Software package, Genetic engineering, DNA

## Abstract

Oligonucleotide synthesis is vital for molecular experiments. Bioinformatics has been employed to create various algorithmic tools for the in vitro synthesis of nucleotides. The main approach to synthesizing long-chain DNA molecules involves linking short-chain oligonucleotides through ligase chain reaction (LCR) and polymerase chain reaction (PCR). Short-chain DNA molecules have low mutation rates, while LCR requires complementary interfaces at both ends of the two nucleic acid molecules or may alter the conformation of the nucleotide chain, leading to termination of amplification. Therefore, molecular melting temperature, length, and specificity must be considered during experimental design. POSoligo is a specialized offline tool for nucleotide fragment synthesis. It optimizes the oligonucleotide length and specificity based on input single-stranded DNA, producing multiple contiguous long strands (COS) and short patch strands (POS) with complementary ends. This process ensures free 5′- and 3′-ends during oligonucleotide synthesis, preventing secondary structure formation and ensuring specific binding between COS and POS without relying on stabilizing the complementary strands based on Tm values. POSoligo was used to synthesize the linear RBD sequence of SARS-CoV-2 using only one DNA strand, several POSs for LCR ligation, and two pairs of primers for PCR amplification in a time- and cost-effective manner.

## Introduction

Long-stranded DNA can be synthesized through DNA synthesis and assembly^1^. DNA synthesis encompasses chemical synthesis of sequences^2^, phosphodiesterase synthesis^3–5^ of sequences, and photolithographic synthesis^6–9^ using photolithographic techniques and photosensitive vectors to synthesize DNA through UV irradiation and masking. These methods may not be universally available, and direct synthesis techniques face challenges in synthesizing large sequences. The primary challenge involves introducing sequence errors during product synthesis, with error probability positively correlated with sequence length^10–12^. Consequently, sequencing a substantial number of cloned sequences is necessary to minimize error incidence, a cost mitigated by advances in sequencing technology, although the risk of errors remains despite error correction and sequence validation efforts^12–16^. To further diminish error risk, DNA assembly^16–23^ offers advantages in long-strand DNA synthesis. By merging multiple small DNA fragments into longer sequences via polymerase chain reaction (PCR) and ligase reaction (LCR), this method proves efficient in terms of accuracy, cost, and time^1^. Various assembly techniques and their associated software have been published.

Hoover and Lutkovski developed DNAWorks, an automated method for designing and optimizing oligonucleotides for PCR-based gene synthesis^24^. The software accepts DNA or protein sequences as input and designs optimized oligonucleotides to match the codon bias of the chosen host for expression. Gibson et al. designed the Gibson Assembly by creating sequences complementary at both ends and utilizing T5 exonuclease to excise the complementary sequence at the 5′ end, ensuring that the two assembled fragments produce identical sticky ends^22^. The Golden Gate assembly, devised and developed by Carola Engler et al. utilizes IIS-type restriction endonucleases to recognize specific sequences of the target gene and create sticky ends, with ligase attaching multiple fragments to the vector plasmid^21,25^. Jean-Marie Rouillard et al. designed an online tool optimizing the synthesis of long-stranded DNA assemblies through various algorithms^26^. Currently, oligonucleotide design for LCR or PCR-synthesized genes relies on two parameters: ensuring similar thermodynamic properties (i.e. melting temperature) to ensure uniform hybridization during assembly and high specificity of oligonucleotides for the target to avoid incorrect assembly.

Our POSoligo software was based on the POS method^27^, utilizing single-stranded DNA as a template, thereby circumventing the need for specific thermodynamic properties in each segment of double-stranded DNA. Moreover, the short patch chains exhibit high specificity for adjacent template chains, thereby reducing the likelihood of mutations during synthesis. In terms of versatility, this software supports the input of single-stranded DNA and RNA sequences.

## Materials and methods

All oligonucleotides were procured from Sangon Biotech Bioengineering (Shanghai) Co. A comprehensive index of sequences was compiled by replicating the designs outlined in the procedural section.

### Phosphorylation of oligonucleotides

Oligonucleotides (0.1 nmol) underwent phosphorylation at the 5′-end in a PCR tube. The reaction, comprising 3 μL of 10 × PNK buffer (0.5 M Tris/HCl pH 7.6, 0.1 M MgCl_2_, 50 mM DTE), 2 μL of T4 polynucleotide kinase (10 units), 1 μL ATP (1 mM), and 23 μL nuclease-free H_2_O, was conducted at 37 ℃ for 30 min. Subsequently, 70 μL of nuclease-free H_2_O was added, and the reaction was halted by incubating on ice.

### Ligase chain reaction

LCR was conducted to covalently link two adjacent long structured oligonucleotides (COS), thus forming full-length sequences. The LCR comprised 1 μL of phosphorylation reaction product, 2.5 μL of 10 × Taq ligase buffer (0.2 M Tris/HCl pH 7.6, 0.25 M potassium acetate, 0.1 M magnesium acetate, 10 mM NAD + , 10% Triton X-100), 1 μL Taq ligase (10 units), and 19.5 μL nuclease-free H2O in a PCR tube. The reaction was carried out on a MyGene series Peltier thermal cycler MG25 + (LongGene, Hangzhou, China) as follows: 95 ℃ for 5 min, 45 cycles of 95 ℃ for 30 s, 51 ℃ for 20 s, and 45 ℃ for 4 s, with a final incubation at 45 ℃ for 5 h.

### Polymerase chain reaction amplification

A partial double-stranded DNA template was obtained by PCR amplification using the outermost primer. The reaction mixture comprised 1 μL of LCR product, 2.5 μL of dNTPs (2 mM), 1 μL of each primer (0.2 μM), 5 μL of 10 × Pfu DNA polymerase buffer, high-fidelity DNA polymerase Pfu (5 U/μL, Bio-Basic Inc., Ontario, Canada), and 38.5 μL of nuclease-free H2O. The PCR reaction proceeded as follows: 94 °C for 3 min, 30 cycles of 94 °C for 30 s, 55 °C for 30 s, and 72 °C for 2 min, followed by a final extension at 72 °C for 5 min.

### Amplification, cloning and sequencing of synthetic fragments

A 6-well plate was prepared with 3 wells of 293FT cells (including duplicate wells and control), at a density of 1 × 10^6^/mL per well. For electro-transfection, 2 μg of gel-recovered DNA and 100 μL of electro-transfection buffer were added to the electro-transfection cup, mixed well in the X-Porator H1 electro-transfer apparatus, and incubated in the incubator after transfection. RNA was extracted from 3 tubes of cytosol (C1, C2, CON) and reverse transcribed to cDNA. The cDNA served as a template, and RBD-F and RBD-R primers (Table 1) were utilized to amplify the RBD target fragment. The reaction proceeded as follows: 94 °C for 3 min, 30 cycles of 94 °C for 30 s, 55 °C for 30 s, and 72 °C for 2 min, followed by a final extension at 72 °C for 5 min. The DNA clone was ligated into pGEM-T vector, and 2 μL of the ligation product was transformed into JM109. Recombinant plasmids were screened using the blue/white spot selection method, and the recombinant plasmid was extracted from the white colonies and sequenced for analysis.

**Table 1:** SARS-CoV-2 S protein 34 oligonucleotides.

Oligonucleotides	Sequences (5′–3′)	Length (nt)
*P1*	TAGACTGACGCGAATCTGGTTGCGTTGAAT	30
*P2*	GGCTCACCCCATAACACTTAAAGG	24
*P3*	CAATCTTACCTGTTTGGCCCGGTGCAATTT	30
*P4*	TAGTTACCCCCGACTTTGCTATCAAGATT	29
*P5*	GTGCTACCAGCCTGGTATATTTCGGTA	27
*P6*	TGGTAACCAACGCCGTTTGTAGGTTGAAAT	30
*P7*	TCTAGAGGATCTTACTTCTTGGGCCCACAAACC	33
*CP1*	ATACAACGTATGCAATGGGCCAAGCTCATG	30
*CP2*	TAATAACTAGTCAATAATCAATGTCAACAT	30
*CP3*	ATTTACCGTAAGTTATGTAACGCGGAACTC	30
*CP4*	AAGTCCCTATTGGCGTTACTATGGGAACAT	30
*CP5*	GGGGCGTACTTGGCATATGATACACTTGAT	30
*CP6*	ATGTACTGCCAAGTAGGAAAGTCCCATAAG	30
*CP7*	CAAACCGCTATCCACGCCCATTGATGTACT	30
*CP8*	GAAAGTCCCGTTGATTTTGGTGCCAAAACA	30
*CP9*	TAAACGAGCTCTGCTTATATAGACCTCCCA	30
*CMV1*	AAAGGTGTGGGTTTGGATCCGGCCTCGGCCTCTGCATAAATAAAAAAAATTAGTCAGCCATGAGCTTGGCCCATTGC	77
*CMV2*	ATACGTTGTATCCATATCATAATATGTACATTTATATTGGCTCATGTCCAACATTACCGCCATGTTGACATTGATTATTGAC	82
*CMV3*	TAGTTATTAATAGTAATCAATTACGGGGTCATTAGTTCATAGCCCATATATGGAGTTCCGCGTTACATAAC	71
*CMV4*	TTACGGTAAATGGCCCGCCTGGCTGACCGCCCAACGACCCCCGCCCATTGACGTCAATAATGACGTATGTTCCCATAGTAACGCC	85
*CMV5*	AATAGGGACTTTCCATTGACGTCAATGGGTGGAGTATTTACGGTAAACTGCCCACTTGGCAGTACATCAAGTGTATCATATGC	83
*CMV6*	CAAGTACGCCCCCTATTGACGTCAATGACGGTAAATGGCCCGCCTGGCATTATGCCCAGTACATGACCTTATGGGACTTTCCTA	84
*CMV7*	CTTGGCAGTACATCTACGTATTAGTCATCGCTATTACCATGGTGATGCGGTTTTGGCAGTACATCAATGGGCGTG	75
*CMV8*	GATAGCGGTTTGACTCACGGGGATTTCCAAGTCTCCACCCCATTGACGTCAATGGGAGTTTGTTTTGGCACCAAAATC	78
*CMV9*	AACGGGACTTTCCAAAATGTCGTAACAACTCCGCCCCATTGACGCAAATGGGCGGTAGGCGTGTACGGTGGGAGGTCTATATAAGCAGA	89
*CMV10*	GCTCGTTTAGTGAACCGTCAGATCGCCTGGAGACGCCATCCACGCTGTTTTGACCTCCATAGAAGACACCGACTCTAGAG	80
*RBD-1*	ATGCGCGTACAACCGACGGAGAGTATCGTACGATTCCCTAACATAACGAATCTCTGTCCGTTTGGAGAGGTATTCAACGCAACCAGATTCGC	92
*RBD-2*	GTCAGTCTATGCGTGGAATCGGAAGAGAATATCTAATTGTGTTGCTGACTATTCTGTGCTGTATAACTCAGCCTCCTTTAGTACCTTTAAGTGTTATG	98
*RBD-3*	GGGTGAGCCCGACAAAACTTAACGACCTTTGCTTTACCAACGTGTACGCCGACAGTTTTGTAATCAGGGGGGATGAAGTTAGGCAAATTGCACCGGGCC	99
*RBD-4*	AAACAGGTAAGATTGCAGACTATAACTACAAATTGCCAGATGACTTCACTGGTTGTGTTATCGCGTGGAATTCTAACAATCTTGATAGCAAAGTCGG	97
*RBD-5*	GGGTAACTATAACTATCTTTACCGCCTGTTTAGAAAAAGTAACCTTAAACCGTTCGAGCGAGACATAAGTACCGAAATATACCAG	85
*RBD-6*	GCTGGTAGCACACCTTGCAATGGGGTGGAGGGGTTCAACTGTTACTTCCCCCTCCAAAGTTATGGATTTCAACCTACAAACGG	83
*RBD-7*	CGTTGGTTACCAGCCTTACAGGGTCGTTGTACTCAGTTTCGAGTTGCTTCATGCTCCTGCTACGGTTTGTGGGCCCAA	78
*RBD-8*	GAAGTAAGATCCTCTAGAAATAAAAGATCTTAAGTTTCATTAGATCTGTGTGTTGGTTTTTTGTGTG	67
*CMV-F*	AAAGGTGTGGGTTTGGATCCGGCCTCGGCCTC	32
*CMV-R*	CTCTCCGTCGGTTGTACGCGCATCTCTAGAGTCGGTGTCTTCTATGGAGG	50
*RBD-F*	ATGCGCGTACAACCGACGGAGAGTATCGTACGATTCCC	38
*RBD-R*	CACACAAAAAACCAACACAC	20

### Algorithm

Our software employs a sophisticated algorithm to convert the input sequence into oligonucleotides. This input sequence is interpreted as single-stranded DNA, which is then divided into consecutive short strands of 50–120 bps.

Additionally, an auxiliary complementary patch strand is calculated and designed to link the two terminal points of COS, serving as a nexus or bridge. Notably, the two terminal regions of the original sequence intentionally lack patch strands, preserving their accessibility.

Following LCR, primers are carefully crafted in the terminal region of the long chain to facilitate PCR amplification.

### Implementation

**Figure 1:**
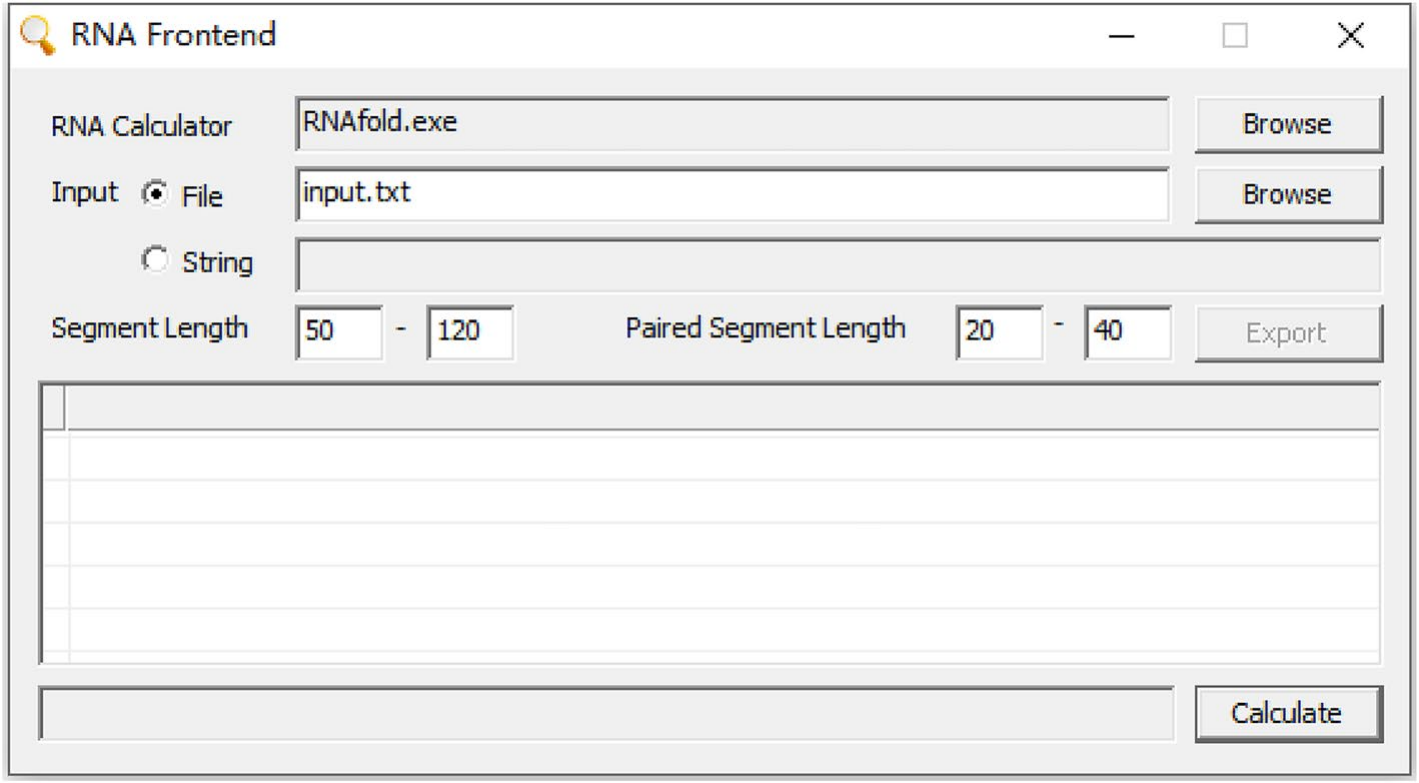
The interface of the POSoligo program enables users to select a document or input a sequence directly into the "Input" section. Users can adjust the lengths of contiguous long strands (COS) and short patch strands (POS) based on the experiment's objectives and requirements. Clicking on "Calculate" generates the results.

POSoligo has been developed using C +  + programming and can be accessed either via direct input sequences or through a .TXT file within the software (Fig. 1). The algorithmic process for POS primarily involves designing a series of overlapping patches, aligning them based on their common sequences, and then merging them to generate the final DNA sequence. This iterative process ensures a satisfactory outcome. The C +  + programming language employs various advanced algorithms and techniques to optimize this process:

- Sequence alignment algorithms identify suitable target sequences for amplification or detection.
- Primer/probe design algorithms select appropriate, specific, and efficient oligonucleotide sequences.
- Secondary structure prediction algorithms prevent non-specific binding or unwanted interactions between oligonucleotides.
- Simulated annealing or genetic algorithms optimize chemical synthesis and minimize errors or side reactions. C +  + programs generate optimized protocols for the design and synthesis of oligonucleotides by integrating these algorithms and techniques.

### Application

#### Design of an oligonucleotide set for SARS-CoV-2

**Figure 2:**
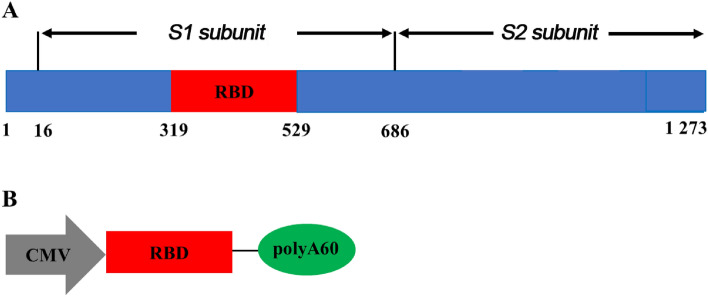
SARS-CoV-2 S protein primary structure and promoter CMV + target antigen RBD + polyA60 complete expression frame. (**A**) RBD located on the S1 subunit in S protein; (**B**) promoter CMV + target antigen RBD + polyA60 complete expression frame.

During the coronavirus pandemic, we developed this software to combat the virus, aiming to save time and streamline PCR synthesis. To evaluate POSoligo, we designed the nucleotide sequence of the coding region of the S1 protein gene of the SARS-CoV-2 virus (GenBank registry no. QHD43416) encompassing the RBD amino acids Arg319–Lys529, totaling 210 base pairs. Subsequently, we appended the CMV promoter at its 5′-end and the polyA tail at its 3′-end to construct the CMV + RBD + polyA60 expression frame (Fig. 2).

**Figure 3:**
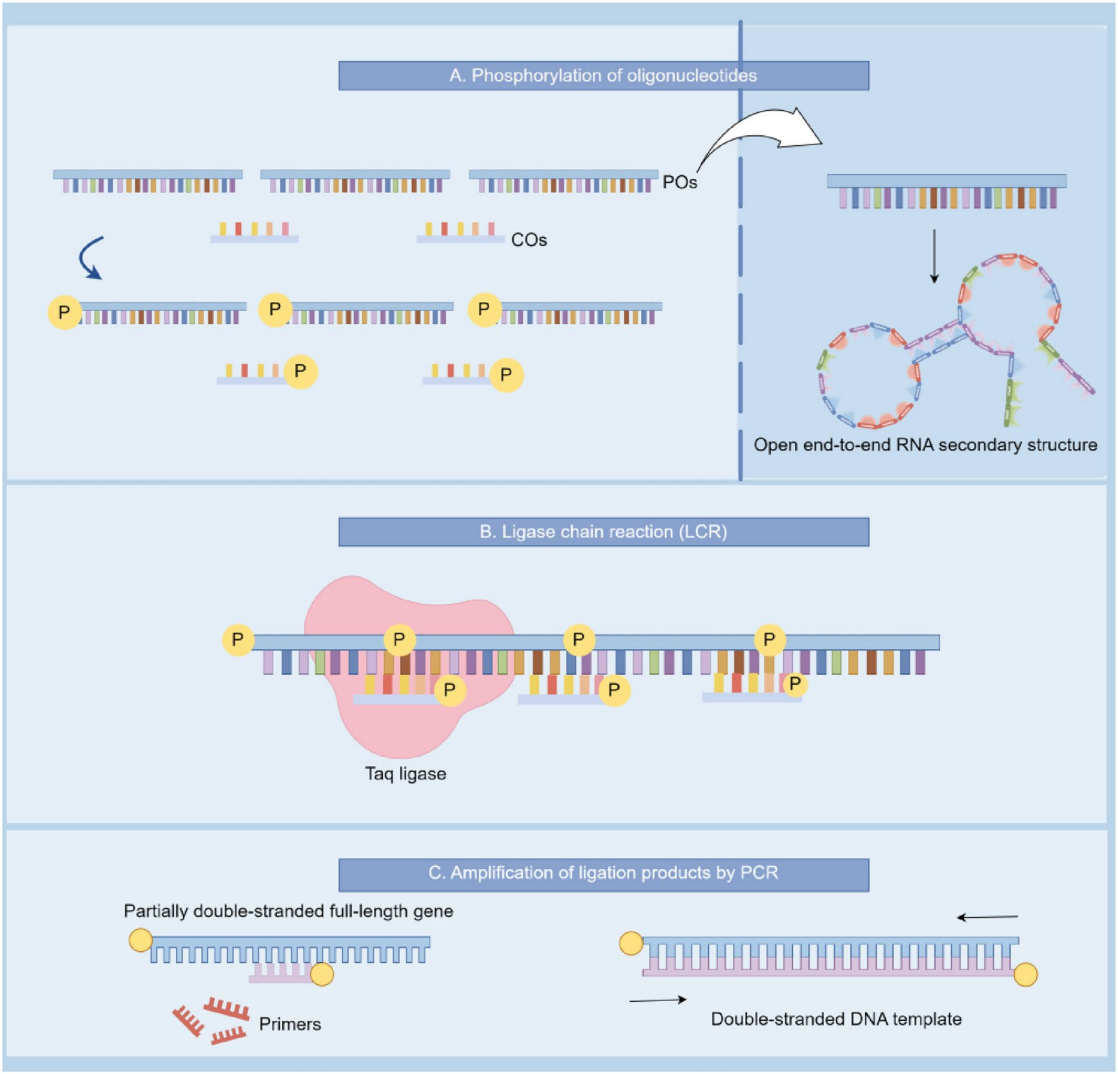
Schematic diagram illustrating the flow of the POS method, with the secondary structure of the POS chain maintaining double ends free in the upper right corner.

The program generated a total of 34 oligonucleotides (Table 1), including CMV1–CMV10 and RBD1–RBD8 as long-structured oligonucleotides (COS) ranging from 50–120 base pairs, and P1–P7 and CP1–CP9 as short structured POS spanning 22–30 base pairs, respectively. Notably, CMV-R served as an intermediate POS for binding the CMV promoter to the RBD reading frame, while CMV-F and RBD-R were utilized as upstream and downstream primers for PCR amplification post-segment assembly. Additionally, RBD-F and RBD-R functioned as primers for reverse transcription cDNAs, followed by sequencing to validate the correct RBD sequence expression. Furthermore, the software's folder was utilized to predict the secondary structure of COS RNA, ensuring free 5′- and 3′-ends, while minimizing self-annealing in the middle portion, which readily denatures at high temperatures (Fig. 3).

#### Synthesis of SARS-CoV-2 *RBD* gene in vitro

CMV1-CMV10, RBD1-RBD8, P1-P7, CP1-CP9, and CMV-R (Table 1) were combined using the 2 × Pfu PCR Mix system and subjected to LCR on MG25 + under the following conditions: 95 °C for 5 min, 45 cycles of 95 °C for 30 s, 51 °C for 20 s, and 45 °C for 4 min, with a final overnight incubation at 45 °C for high-temperature ligation.

The PCR reaction mixture comprised 1 μL of LCR product, 1 μL of each CMV-F and RBD-R primer (Table 1), 12.5 μL of 2 × Pfu PCR Mix, and 9.5 μL of ddH2O. PCR amplification followed this program: 94 °C for 3 min, 30 cycles of 94 °C for 30 s, 55 °C for 30 s, and 72 °C for 2 min, with a final extension at 72 °C for 5 min. Target bands were identified via 1% gel electrophoresis and excised using a gel recovery kit.

**Figure 4:**
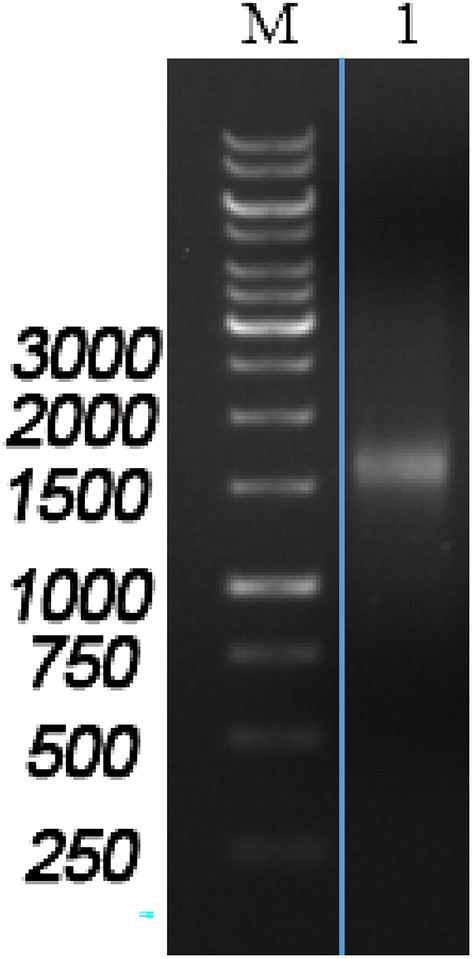
1.0% agarose gel electrophoresis to verify 1 μL LCR-PCR product. M: Marker (1 kbp); 1: LCR-PCR product.

Figure 4 (S1) illustrates the successful in vitro synthesis and amplification of the expression frame sequence of CMV + target antigen RBD + polyA60 (1530 bp) following LCR + PCR reaction. The electrophoresis fragment matched the expected size, confirming the successful synthesis of the expression frame sequence.

#### Synthesis and validation of *RBD* gene

**Figure 5:**
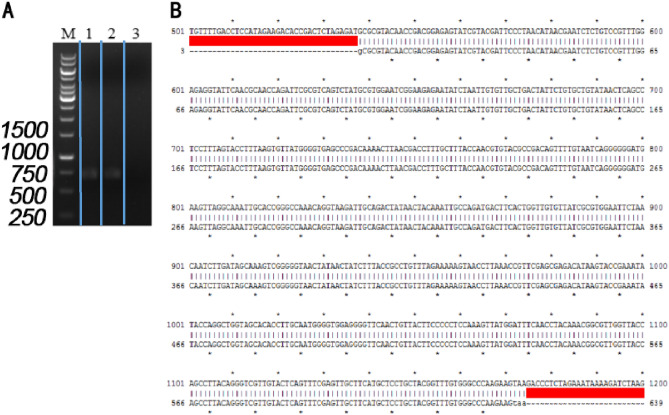
(**A**) 1.0% agarose gel electrophoresis to verify 1 μL cDNA-PCR product. M: Marker (1 kbp); 1, 2: RBD fragments (C1, C2) amplified with reverse transcribed cDNA as the template; 3: negative control (CON). (**B**) Sequencing results.

The LCR-PCR product was recovered from the gel, and its concentration was measured spectrophotometrically, yielding 0.32 μg/μL. Subsequently, the DNA recovered from the gel was transfected into 293FT cells and incubated for 48 h. The RNA extracted and measured spectrophotometrically at a concentration of 1.3 μg/μL was reverse-transcribed into cDNA. The RT-PCR products were analyzed by electrophoresis, showing a match with the expected size of the RBD target gene fragment (Fig. 5A & S2). Sequencing results confirmed the successful synthesis of the RBD fragment, measuring 639 bp (Fig. 5B & Supplement 3).

## Discussion

Our in vitro gene synthesis approach offers numerous advantages compared to many current methodologies and tools. In our previous gene synthesis process, designing a large number of primers using the PCR synthesis method often led to mismatches in repetitive sequences, resulting in shifting or loss of some repetitive sequences in the synthesized whole gene fragment. To reduce the error rate associated with manual primer design for whole gene synthesis, our software generates primers in batch, significantly reducing the mismatch rate and greatly facilitating whole gene synthesis.

Advantage 1: Unlike most modern techniques that require dividing target nucleotides into stable and homogeneous oligonucleotides with consistent thermodynamic properties, such as Gene2Oligo^26^ and Assembly PCR Oligo Maker^28^, our software cleaves sequences into varying lengths while specifically maintaining the 5′- and 3′-ends of each POS sequence free and unobstructed. This design greatly facilitates the correct ligation of adjacent structural oligonucleotides by Taq DNA ligase. Unlike techniques that rely on maintaining a consistent Tm value among different oligodeoxynucleotides, our method releases any residual secondary structure during denaturation at 95 °C in the LCR without considering the stability of the Tm value. While Stemmer et al.^18^ synthesized a 2.7-kbp sequence in a single step, verification during an intermediate stage is necessary due to an increased risk of errors in long sequences, reducing the likelihood of obtaining an accurate fragment after assembly^10–12^. Therefore, shortening long sequences is crucial to improve assembly accuracy. In POSoligo, users can calculate increasingly uniform lengths of oligonucleotides within a defined range using the "segment length" option. Advantage 2: Unlike many current tools that design complete double-stranded DNA using various methods involving special ends, enzymatic methods, or considering different lengths of isolated fragments, our software streamlines the process. It avoids the steep learning curve associated with these tools and instead follows common assembly methods such as PCR and LCR^21,22,25,26,29–32^.

POSoligo provides clear output labeling below the sequence alignment once the calculation is completed. Additionally, it generates an "output" file in the root folder, which can be easily viewed using Notepad or similar software. The results can be copied directly into an email or other applications, facilitating communication with the manufacturer of the synthesized sequence. We have also compiled the program in C +  + to ensure usability across multiple systems, and we plan to develop a web version of the program in the near future to further enhance accessibility.

POSoligo is highly versatile and can be widely used for designing long DNA fragments in synthetic biology and biotechnology research. We are committed to continuously upgrading the functionality of the software to better serve the needs of researchers.

### Supplementary Information

Supplementary Information 1.

Supplementary Information 2.

Supplementary Information 3.

## Data Availability

Data is provided within the manuscript or supplementary information files. The software during the current study are available in the [figshare] repository, DOI: https://doi.org/10.6084/m9.figshare.24879006.v1.
