# Supplementary material for: POSoligo software for in vitro gene synthesis
Source: Sci Rep. 2024 May 15;14:11117. doi: 10.1038/s41598-024-59497-3 (PMC11096389; doi:10.1038/s41598-024-59497-3)
Supplement: Supplementary file 3 — Supplementary Information 3. [file 41598_2024_59497_MOESM3_ESM.pdf]

Supplement

S1: 1.0% agarose gel electrophoresis to verify 1 μL LCR-PCR product.
M: Marker (1 kbp); 1-6: LCR-PCR product. Gel electropherograms of 6 sets of products were shown with empty space between Marker and 5 to avoid overbrightening of the products.

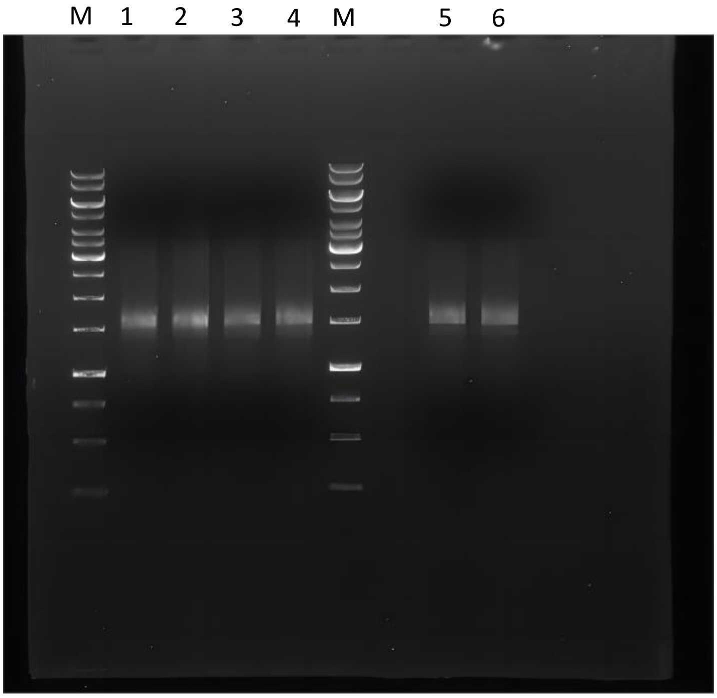

S2:1.0% agarose gel electrophoresis to verify 1 μL cDNA-PCR product.
M: Marker (1 kbp); 1-8: RBD fragments amplified with reverse transcribed cDNA as the template; 9: negative control (CON).

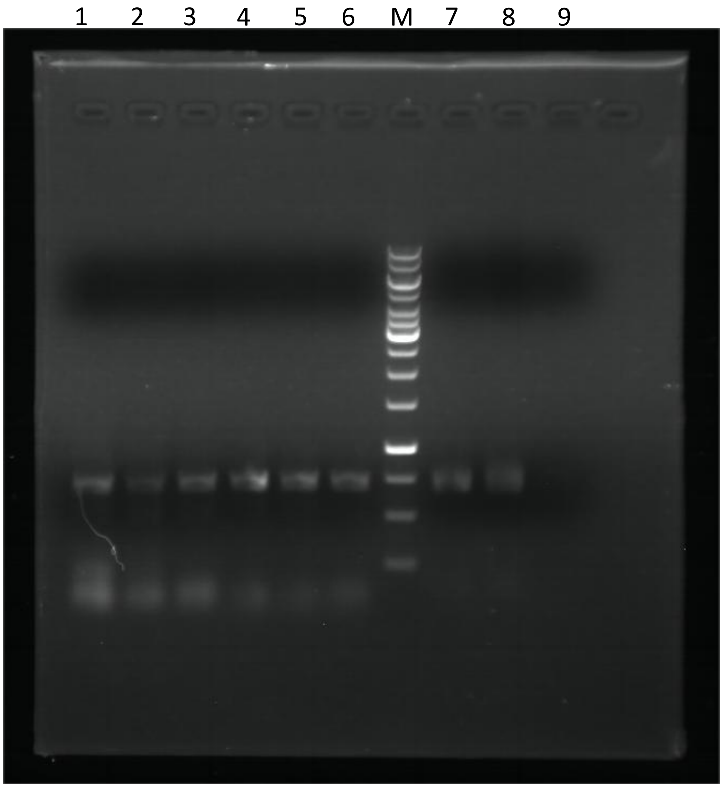

S3: RBD fragment sequencing waveform files are placed in the zip file.
